# Low expression of galectin-3 is associated with poor survival in node-positive breast cancers and mesenchymal phenotype in breast cancer stem cells

**DOI:** 10.1186/s13058-016-0757-6

**Published:** 2016-09-29

**Authors:** Matthias Ilmer, Nachman Mazurek, Michael Z. Gilcrease, James C. Byrd, Wendy A. Woodward, Thomas A. Buchholz, Kim Acklin, Karen Ramirez, Margarete Hafley, Eckhard Alt, Jody Vykoukal, Robert S. Bresalier

**Affiliations:** 1 Department of Translational Molecular Pathology, MD Anderson Cancer Center (MDACC), University of Texas Health Science Center at Houston, Houston, TX USA; 2 Department of Gastroenterology, Hepatology and Nutrition, MD Anderson Cancer Center (MDACC), University of Texas Health Science Center at Houston, Houston, TX USA; 3 Department of Pathology, MD Anderson Cancer Center (MDACC), University of Texas Health Science Center at Houston, Houston, TX USA; 4 Department of Breast Radiation Oncology, MD Anderson Cancer Center (MDACC), University of Texas Health Science Center at Houston, Houston, TX USA; 5 Department of Stem Cell Transplantation and Cellular Therapies, MD Anderson Cancer Center (MDACC), University of Texas Health Science Center at Houston, Houston, TX USA; 6 Department of Medicine, Tulane University Health Science Center, New Orleans, LA USA; 7 Current address: Department of General, Visceral and Transplantation Surgery, Hospital of the University of Munich (LMU), Munich, Germany

**Keywords:** Galectin-3 (Gal3), Cancer stem cells (CSC), Epithelial-mesenchymal transition (EMT), Breast cancer

## Abstract

**Background:**

Galectin-3 (Gal3) plays diverse roles in cancer initiation, progression, and drug resistance depending on tumor type characteristics that are also associated with cancer stem cells (CSCs). Recurrence of breast carcinomas may be attributed to the presence of breast CSCs (BCSCs). BCSCs exist in mesenchymal-like or epithelial-like states and the transition between these states endows BCSCs with the capacity for tumor progression. The discovery of a feedback loop with galectins during epithelial-to-mesenchymal transition (EMT) prompted us to investigate its role in breast cancer stemness.

**Method:**

To elucidate the role of Gal3 in BCSCs, we performed various in vitro and in vivo studies such as sphere-formation assays, Western blotting, flow cytometric apoptosis assays, and limited dilution xenotransplant models. Histological staining for Gal3 in tissue microarrays of breast cancer patients was performed to analyze the relationship of clinical outcome and Gal3 expression.

**Results:**

Here, we show in a cohort of 87 node-positive breast cancer patients treated with doxorubicin-based chemotherapy that low Gal3 was associated with increased lymphovascular invasion and reduced overall survival. Analysis of in vitro BCSC models demonstrated that Gal3 knockdown by small hairpin RNA (shRNA) interference in epithelial-like mammary spheres leads to EMT, increased sphere-formation ability, drug-resistance, and heightened aldefluor activity. Furthermore, Gal3^negative^ BCSCs were associated with enhanced tumorigenicity in orthotopic mouse models.

**Conclusions:**

Thus, in at least some breast cancers, loss of Gal3 might be associated with EMT and cancer stemness-associated traits, predicts poor response to chemotherapy, and poor prognosis.

**Electronic supplementary material:**

The online version of this article (doi:10.1186/s13058-016-0757-6) contains supplementary material, which is available to authorized users.

## Background

Breast cancer is the leading cause of cancer-related deaths in women [1]. Advances in the treatment of this heterogeneous group of diseases are therefore a high priority. One approach is to detect and selectively target cancer stem cells, a small subset of the tumor cell population [2]. The cancer stem cell hypothesis suggests that many cancers, including breast cancer, are driven by a small subpopulation of cells that displays stem cell properties. These cells are rare, highly heterogeneous in nature, and characterized by their tumorigenic potential and unlimited self-renewal capability. They may mediate tumor progression and, by virtue of their relative resistance to chemotherapy and radiation, contribute to treatment relapse [3, 4]. The definitive identification of clinically relevant breast cancer stem cell (BCSC) subgroups that ultimately reduce patient survival, however, remains a challenge. Recent studies have indicated a close association between BCSCs and the acquisition of an epithelial–mesenchymal transition (EMT) state [5].

BCSCs have been shown to exist in distinct mesenchymal-like (EMT) and epithelial-like (mesenchymal–epithelial transition; MET) states [6]. Mesenchymal-like BCSCs have been characterized as CD24^negative^/CD44^positive^ and appear to be primarily quiescent and localized at the tumor invasive front. Epithelial-like BCSCs in contrast tend to be proliferative and located more centrally. The process of EMT plays an important role in embryogenesis as well as in a number of biological processes associated with cancer progression [7]. During EMT, epithelial cells lose cell–cell contacts, undergo cytoskeletal remodeling resulting in loss of polarity, and acquire a mesenchymal morphology [8]. Interestingly, a number of pathways that are known to regulate BCSC, including canonical and noncanonical Wnt signaling or transforming growth factor beta (TGFβ) pathways, are also capable of inducing EMT [9]. However, other pathways, such as the human epidermal growth factor receptor (HER) signaling, promote MET [10]. Although several BCSC markers have been described, it is unclear whether these markers identify the same or distinct BCSCs.

Galectins are carbohydrate-binding proteins characterized by their binding affinity for β-galactosides and by conserved sequences in the carbohydrate-binding site [11]. It has been shown that galectin-3 (Gal3) is responsible for a myriad of biological processes in a wide variety of cancers. In breast cancers, Gal3 expression may be associated with specific morphological precursor subtypes and undergoes a transitional shift in expression from luminal to peripheral cells as tumors progress to comedo-type ductal carcinoma in situ or invasive carcinomas [12, 13]. Recently, a functional feedback loop between beta1 integrins and galectin-1 that involves the epigenetic induction of galectin-1 expression during integrin-induced EMT and cell scattering was identified [14].

The role of Gal3 CSCs is still controversial. Recently, Chung and colleagues reported in a lung cancer model that Gal3 correlated with tumor progression and increased the CSC pool by activation of the Wnt signaling pathway [15]. It was also suggested that Gal3 could be a therapeutic target in some breast cancers [16].

Gal3 appears therefore to functionally and structurally contribute to a number of BCSC hallmarks, which prompted us to investigate its role in breast cancer stemness.

## Methods

### Cell culture

The cell lines GI-101A and GI-LM2 were kind gifts from Dr. Janet Price and described elsewhere [17]. GI-LM2G are GI-LM2 cells with stable lentiviral knockdown of Gal3 as described below. Cells were maintained and subcultured as explained before [18].

### Reagents

B27 supplement was purchased from Life Technologies, Carlsbad, CA, USA (#17504-044), rhEGF from Sigma-Aldrich, St. Louis, MO, USA (#E9644), and recombinant human fibroblast growth factor (rhFGF) from BD Biosciences, San Jose, CA, USA (#354060).

### Sphere culture and sphere-formation assays

Parental cells were trypsinized, washed with phosphate-buffered saline (PBS), and seeded in clonal density (5–10 × 10^3^/mL) in cancer stem cell (CSC) media consisting of Dulbecco’s modified Eagle’s medium (DMEM) supplemented with L-glutamine, B27, recombinant human epidermal growth factor (rhEGF) (10 ng/mL)/rhFGF (10 ng/mL), and penicillin/streptomycin on ultra-low attachment plates (Corning, Corning, NY, USA). Conditioned media (CM) was changed every 3–4 days and spheres trypsinized and reseeded for expansion in higher generations. For quantification of sphere-formation ability, single cell suspensions were seeded into ultra-low attachment 96-well plates (500–1000 cells/100 μL) in CM plus 1 % methylcellulose. One hundred microliters of the same media was added after 3 days and exchanged after another 3–4 days. Spheres were usually counted after 10 days. Only spheres >75 μm in diameter were included, if not otherwise stated.

### shRNA silencing of galectin-3

Cells were infected with the galectin-3 shRNA (#sc-155994-V) and nontargeting control shRNA (#sc-108080) lentiviral particles (Santa Cruz Biotechnology, Dallas, TX, USA) according to the manufacturer’s protocol. After transduction, stable cell lines expressing the galectin-3 (or control) shRNA were isolated by selection with 2.5 pg/ul puromycin (Santa Cruz Biotechnology; #sc-108071A). Protein expression by Western blot analysis or cell surface expression by flow cytometry was verified following propagation for four passages.

### Aldehyde dehydrogenase (ALDH) assay

Spheres were dissociated into single-cell suspension and allowed to recover for 24 h in CSC media in low-attachment plates. ALDH activity was monitored using the ALDEFLUOR kit (Stemcell Technologies, Vancouver, BC, Canada; #01700) following manufacturer’s instructions. The ALDH activity was analyzed by flow cytometry using the LSRFortessa and quantified by FlowJo 8.8.6 software, Multicycle cell software (FlowJo LLC., Ashland, OR, USA).

### Luciferase reporter assays

CSC cells were cultured in CSC medium in triplicate in low-attachment 24-well plates. Lipofectamine 2000 (Life Technologies; #11668-027) was used to transiently transfect cells with either 500 ng inducible Firefly luciferase expressing either SuperTOP or SuperFOP vector (AddGene, Cambridge, MA, USA; plasmids #12456 and #12457) for monitoring Wnt signaling or SBE4luc (AddGene plasmid #16495) for monitoring TGFβ signaling. Simultaneously, cells were cotransfected with constitutively Renilla luciferase expressing normalization control vector pRL-TK (Promega, Madison, WI, USA; #E2241)) at a ratio of 50:1. Twenty-four hours after transfection, Firefly and Renilla luciferase activity were measured using Dual-Glo Luciferase Reporter Assay System (Promega; #E2920) and a microplate reader (Dynex Technologies, Chantilly, VA, USA). The Renilla luciferase activity was normalized to the Firefly luciferase activity. All experiments were performed in triplicate.

### Immunohistochemistry (IHC) and immunofluorescence (IF)

Immunohistochemical and immunofluorescence stainings were carried out as described before [19]. Briefly, paraformaldehyde (PFA)-fixed and paraffin-embedded tissue sections underwent antigen retrieval in sodium citrate buffer (pH 6.0) at boiling temperature for 20 minutes. Sections were washed in Tris-buffered saline (TBS) plus 0.025 % Triton X-100 after cooling down, blocked with 10 % goat serum, and incubated with primary antibody solutions at 4 ° C overnight. After several washes, slides were directly incubated with secondary antibody solution at room temperature for 1.5 hours in the dark. Immunofluorescent slides were counterstained with 4,6-diamidino-2-phenylindole (DAPI) and then mounted in Fluorescence Mounting Medium (Dako, Glostrup, Denmark). IHC sections were stained using the VECTASTAIN Elite ABC Kit (Universal) according to the manufacturer's instructions (Vector Laboratories, Burlingame, MA, USA). For primary goat antibodies, we used a horseradish peroxidase-conjugated anti-goat secondary antibody solution.

Cytoplasmic staining was scored by multiplying an intensity score (0 = negative, 1 = weak, 2 = moderate, and 3 = strong) × the percentage of invasive tumor cells with staining. A score of 150 or greater was considered high expression.

### In vivo xenograft experiments

Mouse experiments were performed in accordance with the Institutional Animal Care and Use Committee (IACUC) at M.D. Anderson. Immunocompromised age-matched male 5- to 6-week-old, athymic, nu/nu, Balb/c mice were purchased from an institutional breeder colony and kept at 24 °C in sterile conditions with water and food ad libitum. Different dilutions of GI-LM2 or GI-LM2G cells were orthotopically implanted in 50 μL PBS into the fourth mammary fat pad via a 27G needle. Tumor growth was controlled and measured regularly and mice were checked for their vital status and weight. Tumor volume was calculated by the following formula: mm^3^ = (width × width × length) / 2 [mm]. All mice were sacrificed when their tumor volumes reached high tumor burdens according to the IACUC protocol or at the end of the experiment. Subsequently, tumor parts that were selected for further in vitro cell culture experiments were fixed in 4 % PFA for histological analyses.

### Patients

This study was approved by The University of Texas M.D. Anderson Cancer Center institutional review board. Patients included in this retrospective study were treated - after obtaining the necessary consent - on Protocol DM86-12, a randomized study comparing six cycles of 5-fluorouracil (5-FU), doxorubicin (A), and cyclophosphamide (C) in the adjuvant setting to six cycles of fluorouracil, doxorubicin, and cyclophosphamide followed by four cycles of methotrexate and vinblastine. Although patients ≥50 y of age with estrogen receptor-positive disease were randomized to receive tamoxifen or six cycles of fluorouracil, doxorubicin, and cyclophosphamide plus four cycles of methotrexate and vinblastine, those who received tamoxifen were excluded from our retrospective study, so all patients in our study received doxorubicin-based chemotherapy without tamoxifen. The previous clinical protocol failed to show any benefit from the addition of four cycles of methotrexate and vinblastine to six cycles of fluorouracil, doxorubicin, and cyclophosphamide, so both groups were regarded as having similar doxorubicin-based chemotherapy.

Inclusion criteria for this retrospective study were resectable stages II and IIIA breast cancer with axillary lymph node metastases, surgical treatment with mastectomy and axillary dissection without irradiation, age younger than 75 y at diagnosis, no evidence of distant disease at diagnosis, and no history or concurrent malignancy. Additional entry criteria included availability of sufficient archival paraffin-embedded tumor tissue from the primary breast tumor to obtain cores for tissue microarrays. All patients had surgery done at M.D. Anderson Cancer Center between 1986 and 1994.

### Tissue microarrays

To facilitate the efficient use of patient specimens, tissue microarrays (TMA) were constructed. Paraffin blocks containing tissue from the primary breast tumor were used. The TMAs were prepared using a manual tissue puncher/array (Beecher Instruments, Sun Prairie, WI, USA). Up to six cores, 0.6 mm in diameter, were cut from each primary tumor and aligned within the recipient block in a rectilinear array. All cores were placed 0.2 mm apart in the recipient blocks.

### Statistical analyses

For the analyses of TMA results and patient outcome data, locoregional recurrence-free (LRRF), disease-specific (DSS) and overall survival (OS) estimates were calculated using the Kaplan-Meier product limit method and were expressed ± standard error. The two-sided log-rank test was used to test the association between particular factors and patient survival. All statistical analyses were carried out using SSPS 12.0 for Windows (SPSS Inc., Chicago, IL, USA). Locoregional recurrence-free survival was defined as the interval from the date of surgery to the date of first locoregional disease recurrence or to the last follow-up date. All locoregional recurrences were scored as events regardless of the presence of distant metastatic disease, and patients without recurrence were censored at last follow-up. Disease-specific survival was defined as the interval from the date of surgery to the date of death due to breast cancer. Overall survival was defined as the interval from the date of surgery to the date of death from any cause.

Results are expressed as the mean ± standard error of the mean (SEM). All statistical comparisons for the in vitro and mouse data were made with a standard *t* test, using biostatistics software from GraphPad Prism® (La Jolla, CA, USA). The criteria for significance were *p* < 0.05 (*), *p* < 0.01 (**), and *p* < 0.001 (***) for all comparisons. Additional methods can be found in the Additional file 1.

## Results

### Gal3 expression, clinicopathological features, and survival

**Fig. 1:**
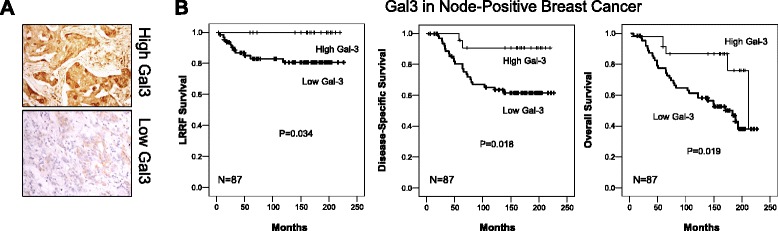
Low galectin-3 (Gal3) expression correlates with poor outcome in node-positive breast cancer. **a** Representative pictures of IHC staining for Gal3 in TMAs with examples for Gal3 high (*upper panel*) and Gal3 low (*lower panel*). **b** Tumor microarrays (TMA) of node-positive breast cancer patients were stained for Gal3. High Gal3 expression shows better locoregional relapse-free (LRRF) survival (*left panel*), better disease-specific survival (DSS) (*middle panel*) as well as better overall survival (OS) (*right panel*) than low Gal3 expression in node-positive breast cancer

To assess the clinical relevance of Gal3 expression and BCSC features, we investigated a cohort of 87 node-positive breast cancer patients treated with doxorubicin-based chemotherapy (FAC; 5-fluorouracil, adriamycin, cyclophosphamide).) with respect to tumor recurrence and survival. Tissue microarrays (TMAs) were prepared from the primary tumors and stained for Gal3 (Fig. 1a). The clinicopathological variables of the patients in this cohort are illustrated in Table 1. No association of Gal3 expression with any particular morphology or distribution pattern was evident in these tissue microarray specimens. The subcellular distribution of Gal3 was generally cytoplasmic. Only three of the tumors had nuclear expression, which was too few for meaningful analysis. Cytoplasmic staining was evaluated by H-score classification, where a score of 150 or greater was considered high expression. We could not detect any statistically significant difference in Gal3 expression with respect to age (*p* = 0.41), race (*p* = 0.25), menopausal status (*p* = 0.57), number of lymph node metastasis (*p* = 0.36), or hormone receptor status (ER *p* = 0.56; PR *p* = 0.37; HER2 *p* = 0.21). However, we found that the lymphovascular invasion significantly correlated with low Gal3 expression (*p* = 0.01) (Table 1).

**Table 1:** Gal3 expression according to clinicopathologic variables

Variable	Total patients	High Gal3	Low Gal3
Age		*p* = 0.41	
20–35 yr	7	2	5
36–50 yr	48	12	36
51–70 yr	31	9	22
> 70 yr	1	0	1
Race		*p* = 0.25	
Black	7	3	4
White	61	18	43
Hispanic	14	2	12
Other	5	0	5
Menopausal status		*p* = 0.57	
Pre	47	13	34
Post	37	10	27
Unknown	3	0	3
pT stage		*p* = 0.22	
pT1 (0–2 cm)	22	9	13
pT2 (2–5 cm)	55	13	42
pT3 (>5 cm)	7	1	6
Unknown	3	0	3
Tumor grade		*p* = 0.41	
1	5	2	3
2	38	12	26
3	44	9	35
pN stage		*p* = 0.36	
N1 (1–3 LN)	60	16	44
N2 (4–9 LN)	19	5	14
N3 (>9 LN)	8	2	6
Lymphovascular invasion		*p* = 0.01	
Present	38	5	33
Absent	49	18	31
ER		*p* = 0.56	
Positive	53	13	40
Negative	33	10	23
Unknown	1	0	1
PR		*p* = 0.37	
Positive	40	9	31
Negative	45	14	31
Unknown	2	0	2
HER2		*p* = 0.21	
0	48	13	35
1+	13	2	11
2+	5	0	5
3+	20	8	12
Unknown	1	0	1

*Gal3* galectin-3, *ER* estrogen receptor, *HER2* human epidermal growth factor receptor 2, *PR* progesterone receptor

Subsequent univariate analysis revealed that low Gal3 expression is associated with decreased locoregional recurrence-free, disease-specific and overall survival (*p* = 0.034, *p* = 0.18, and *p* = 0.019, respectively) (Table 2 and Fig. 1b). Multivariate analysis demonstrated statistically significant correlations with ≥10 positive lymph nodes (*p* = 0.020) in locoregional recurrence-free survival, low Gal3 in disease-specific survival (*p* = 0.003) and overall survival (*p* = 0.014), respectively, and HER2 IHC score in locoregional recurrence-free or overall survival (*p* = 0.003 and *p* = 0.001, respectively) (Table 2). Analysis of data from The Cancer Genome Atlas (TCGA) demonstrated that gene expression (*LGALS3*) decreases sequentially from normal tissue to ductal breast carcinoma in situ to invasive ductal breast carcinoma (Additional file 2: Figure S1A). Moreover, it appears that *LGALS3* expression is lower at metastatic sites compared to primary breast tumors (Additional file 2: Figure S1B). Although not statistically significant, we were also able to detect more cases with low Gal3 expression in higher tumor stages (*p* = 0.22) and tumor grades (*p* = 0.41) in our patient cohort (Table 1). Together, these data suggest that lower Gal3 expression is associated with advanced locoregional invasion and poor survival.

**Table 2:** Variables associated with clinical outcome

Locoregional recurrence-free survival (LRRS)			
Univariate		Multivariate	
≥10 positive lymph nodes	*p* = 0.017	≥10 positive lymph nodes	*p* = 0.020
Low Gal3	*p* = 0.034		
Disease-specific survival (DSS)			
Univariate		Multivariate	
Low Gal3	*p* = 0.018	Low Gal3	*p* = 0.003 *p* = 0.003
Her2 IHC score	*p* = 0.008	HER2 IHC score	
Race	*p* = 0.030		
Overall survival (OS)			
Univariate		Multivariate	
Low Gal3	*p* = 0.019	Low Gal3	*p* = 0.014
Her2 IHC score	*p* = 0.015	HER2 IHC score	*p* = 0.001

*Gal3* galectin-3, *HER2* human epidermal growth factor receptor 2, *IHC* immunohistochemistry

### Gal3 expression in isogenic epithelial breast cancer cells

**Fig. 2:**
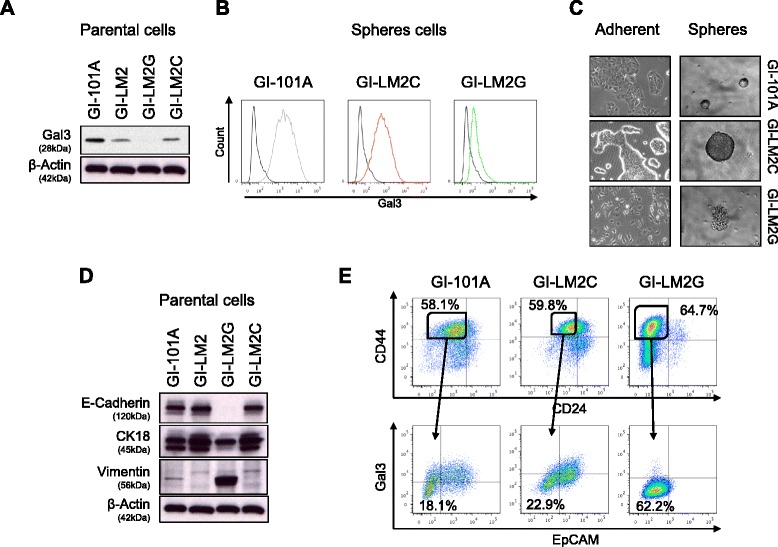
Galectin-3 (gal3) expression is intertwined with an epithelial phenotype. **a** Western blot on Gal3 in the breast cancer cell lines GI-101A, GI-LM2, GI-LM2G, and GI-LM2C. **b** Surface Gal3 expression of spheres was detected by flow cytometry. IgG control is shown in *black*, Gal3 expression of GI-101A (*grey*, *left panel*), Gal3 expression of GI-LM2C (*red*, *middle panel*), and Gal3 expression of GI-LM2G (*green*, *right panel*). **c** Bright field pictures of adherent cells and mammosphere cultures of breast cancer cells used in (**a**). **d** Western blot on EMT markers (E-cadherin, cytokeratin-18 (CK18), vimentin) in breast cancer cells used in (**a**). The same blot is used as in (**a**). **e** Flow cytometry on the cancer stem cell markers CD24, CD44, EpCAM, and Gal3 of parental breast cancer cells. The *upper panel* shows the typically analyzed CD24^neg^/CD44^pos^ CSCs and the *lower panel* displays EpCAM^neg^/Gal3^neg^ populations out of the CD24^neg^/CD44^pos^ CSC pool

To further investigate the mechanisms which explain our findings, we examined isogenic cell lines which differ in their metastatic potential. GI-101A is an estrogen-receptor and EGFR-positive, basal-like low metastatic cell line derived from a primary infiltrating ductile breast tumor [20] and its counterpart GI-LM2 is a highly metastatic variant that was isolated from repeated lung metastasis of GI-101A (Additional file 2: Figure S1C) [17]. Western blot analysis revealed that total Gal3 expression was higher in GI-01A than in GI-LM2 cells (Fig. 2a). Complete depletion of Gal3 by shRNA (delivered by lentiviral particles) in GI-LM2 cells (designated GI-LM2G) was verified by Western blot of whole cell lysates (Fig. 2a). Low surface Gal3 expression was also maintained after culture in anoikis-resistant sphere conditions (Fig. 2b). Gal3 depletion was associated with a striking transition in the morphology of GI-LM2G cells in adherent (Fig. 2c, left panel) as well as sphere conditions (Fig. 2c, right panel). The grape-like phenotype encountered in GI-LM2G spheres corresponds to mesenchymal features in pancreatic cancer cells [19], which prompted us to analyze further EMT markers. GI-LM2G, but not its control cell line GI-LM2C (infected with lentiviral particles harboring a nontargeting shRNA), acquired a mesenchymal-like morphology with spindle-like phenotype whereas GI-101A, GI-LM2 or GI-LM2C formed epithelial-like cell clusters. Moreover, EMT after galectin-3 depletion was further supported by the diminished expression of epithelial markers, E-cadherin and cytokeratin 18 (CK18) and the reappearance of the mesenchymal marker vimentin in GI-LM2G by Western blot analysis (Fig. 2d). EMT features after Gal3 depletion remained stable as evidenced by immunofluorescence staining for the same markers in spheres (Additional file 2: Figure S2A and S2B). Because EMT has been closely connected with cancer stemness in breast cancer, we examined the expression of commonly accepted BCSC surface markers by flow cytometry [5].

Loss of Gal3 was associated with a slightly increased population of CD24^negative^/CD44^positive^ cells (58.1 % in GI-101A vs 64.7 % in GI-LM2G, N.S.) (Fig. 2e, upper panel). When analyzed in greater detail, we found that Gal3^low^-expressing breast cancer cells from all cell lines consistently contained a larger population of BCSC marker-positive subgroups compared to their Gal3^high^ counterparts, (in GI-101A 52.2 % vs. 38.8 %, in GI-LM2C 43.3 % vs. 25.4 % and GI-LM2G 66.2 % vs. 39.7 %) (Additional file 2: Figure S3A). Loss of Gal3 also led to a decreased expression of EpCAM, an epithelial cell adhesion protein, further suggesting a loss of epithelial cell characteristics (Fig. 2e, lower panel and Additional file 2: Figure S3B).

These findings suggest that changes in Gal3 expression lead to alterations of the EMT state in breast cancer cells which remains a conserved hallmark in mammary spheres.

### Loss of Gal3 increases functional CSC characteristics in breast cancer spheres

**Fig. 3:**
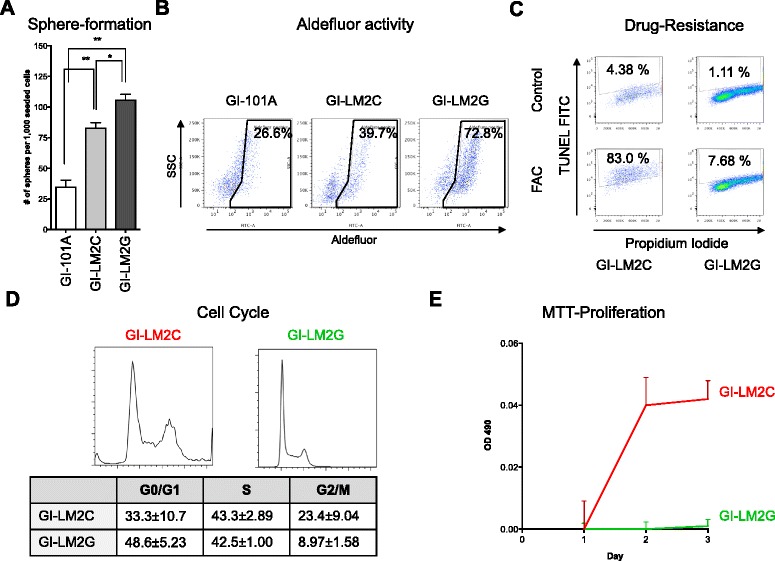
Loss of galectin-3 (Gal3) leads to an increase of functional CSC abilities. **a** Breast cancer cells were seeded into sphere-formation assays (SFA). Loss of Gal3 correlates with the inherent SFA ability displayed as number of spheres per 1000 seeded cells (n = 3). **b** Aldefluor activity of the breast cancer cell lines GI-101A, GI-LM2, and GI-LM2G was determined by flow cytometry. Similar to (**a**), loss of Gal3 increased the percentage of Aldefluor^pos^ cells from 26.6 % (GI-101A) and 39.7 % (GI-LM2) to 72.8 % (GI-LM2G). **c** Drug resistance to the FAC regimen (5-fluorouracil (5-FU), doxorubicin (adriamycin), and cyclophosphamide) was evaluated in CD24^neg^/CD44^pos^ CSCs of GI-LM2C and GI-LM2G by APO-BrDU TUNEL assay (detected by an Alexa Fluor™ 488 dye-labeled anti-BrdU antibody as shown on the y-axis). GI-LM2C undergo cell death upon treatment, whereas GI-LM2G are mostly resistant (83.0 % vs. 7.68 %). **d** Cell cycle distribution was carried out by propidium iodide staining of GI-LM2 (*red*, *left panel*) and GI-LM2G (*green*, *right panel*) and evaluated by flow cytometry. Numbers including standard deviations are shown in the table. **e** MTT proliferation assay of spheres of GI-LM2 (*red*) and GI-LM2G (*green*) over 3 days. The relative OD490 on day 1 was defined as the starting point. Experiments were repeated at least three times. The criteria for significance were *p* < 0.05 (*) and *p* < 0.01 (**)

We next investigated whether loss of Gal3 affects BCSC properties including self-renewal capability, Aldefluor activity, and drug resistance. Sphere-formation assays demonstrated that the cell line with higher metastatic potential (GI-LM2C) formed more than twice as many spheres as low-metastatic GI-101A cells. GI-LM2G cells displayed an even higher sphere-forming capacity compared to GI-LM2C (Fig. 3a and Additional file 2: Figure S4A). This inversely correlated with the Gal3 expression of these cell lines. In line with that, Gal3 depletion in GI-101A led to a significantly enhanced sphere-formation ability (Additional file 2: Figure S4B). Aldefluor activity has been reported to correlate very well with CSC potential in different tumor types [21, 22]. The cell line GI-101A had the lowest (26.6 %), GI-LM2C intermediate (39.7 %) and GI-LM2G the highest Aldefluor activity (72.8 %) (Fig. 3b). FAC (5-fluorouracil (5FU), doxorubicin (adriamycin), and cyclophosphamide) is a widely used chemotherapy regimen used for treatment of early and node-positive breast cancer and was part of the regimen in our patient cohort as well [23]. Exposure of BCSCs to clinically relevant doses of FAC resulted in 83.0 % cell death in GI-LM2C spheres while almost all GI-LM2G spheres were completely resistant (only 7.68 % cell death was observed) (Fig. 3c).

Further, when investigating cell cycle (Fig. 3d) and cell growth of spheres by MTT proliferation assays (Fig. 3e), we found that GI-LM2C spheres displayed significantly higher numbers of cells in G2 phase (23.4 ± 9.04 % vs. 8.97 ± 1.58 %) and grew faster when compared to GI-LM2G spheres. This is in line with observations of Liu et al. that mesenchymal cells are rather quiescent whereas epithelial BCSCs seem to be more proliferative [6].

Collectively, these functional assays demonstrate that Gal3^negative^ breast cancer cells are not only characterized by morphological differences, but also display functional in vitro differences that strongly suggest an association between the absence of Gal3 and BCSC characteristics.

### Canonical Wnt signaling activity and AKT activity are associated with Gal3 expression in epithelial CSC-like spheres

**Fig. 4:**
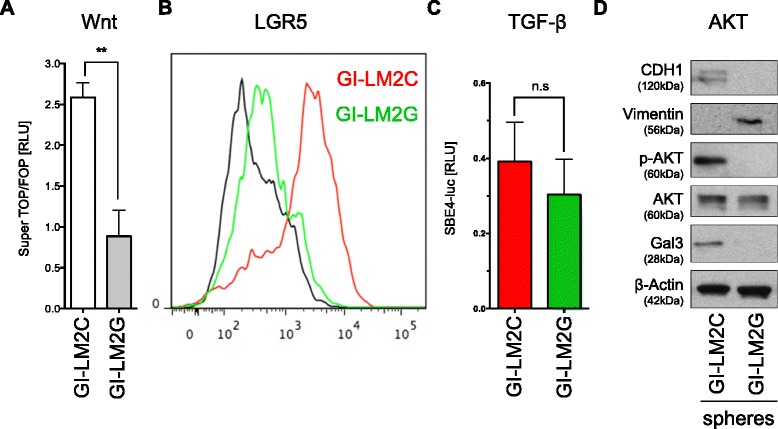
Knockdown of galectin-3 (Gal3) reduces canonical Wnt and AKT signaling in CSC-like spheres. **a** Canonical Wnt signaling activity was evaluated by SuperTOP/FOP (STF) assays and normalized to Renilla luciferase (RLU = relative light units). Baseline Wnt activity was reduced in Gal3^negative^ GI-LM2G spheres (*grey column*) compared to Gal3^positive^ GI-LM2C spheres (*white column*). **b** Flow cytometric analysis of the Wnt target gene LGR5 in GI-LM2C (*red*) and GI-LM2G (*green*) spheres. Canonical Wnt activity in (**a**) correlated with LGR5 expression. **c** TGFβ signaling was evaluated by SBE4-luc assays measured as normalized firefly to Renilla luciferase activity (RLU = relative light units). GI-LM2C (*red column*) and GI-LM2G (*green column*) revealed no significant difference in TGFβ activity. **d** Western blot analysis shows typical results for EMT markers (E-cadherin, vimentin), Gal3 as well as phospho-AKT/total-AKT in GI-LM2C and GI-LM2G spheres. P-AKT was absent in GI-LM2G. Experiments were carried out in triplicate (*n* = 3)

In an attempt to determine their underlying mechanisms with respect to breast cancer stem cell signaling, we analyzed three reported pathways commonly involved in BCSC regulation. Canonical Wnt and TGFβ signaling have recently been linked to lung metastasis in breast cancer cells [24]. We therefore carried out SuperTOP/FOP Luciferase assays (STP) in GI-LM2C and GI-LM2G spheres to analyze the baseline activity of canonical Wnt signaling. Surprisingly, we found that STF activity was higher in Gal3^positive^ GI-LM2C spheres and significantly lower in GI-LM2G spheres (Fig. 4a). These findings are in accordance with previous reports of a positive correlation between Gal3 expression and canonical Wnt signaling [25]. In line with these findings we observed that surface expression of the Wnt target gene, LGR5, was lower in Gal3^negative^ GI-LM2G (green line) than in GI-LM2C spheres (red line) (Fig. 4b) and the protein expression of typical Wnt targets Axin2 and Tcf4 was robustly reduced in GI-LM2G spheres (Additional file 2: Figure S4C).

When examining TGFβ signaling activity, we found no statistically significant difference between GI-LM2C and GI-LM2G as examined by SBE4luc luciferase readouts (Fig. 4c). Strikingly, however, GI-LM2C demonstrated high phospho-AKT activity (p-AKT) indicating activation of the AKT pathway, whereas GI-LM2G did not show any p-AKT on Western blot analysis (Fig. 4d). The latter results are consistent with previous findings showing that Gal3 can activate AKT in bladder cancer cells [26]. AKT pathway activation has also been associated with increased proliferation [27]. Together, we found that activation of AKT and Wnt in Gal3^positive^ epithelial spheres was accompanied by increased cell cycle and heightened proliferation as shown in Fig. 3d and e. These observations support the concept that epithelial-like spheres are proliferative and mesenchymal-like BCSCs are quiescent [6].

### Breast CSCs with low Gal3 expression possess heightened tumorigenicity in vivo

**Fig. 5:**
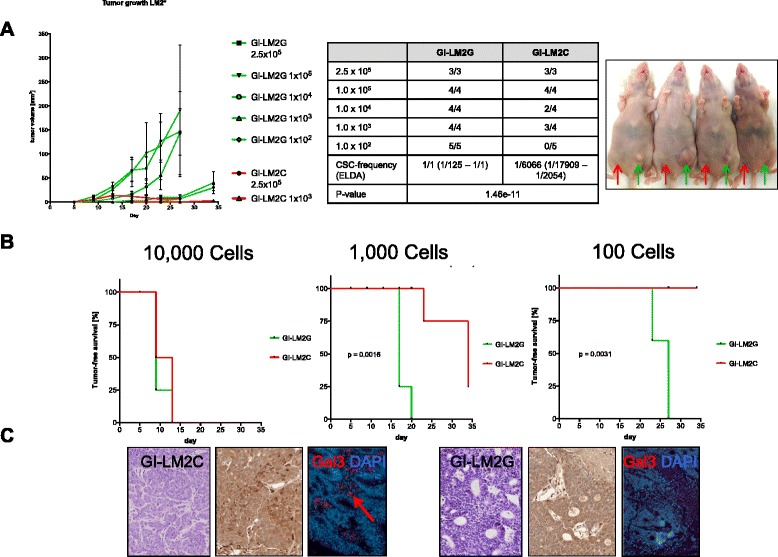
Low galectin-3 (Gal3) correlates with heightened tumorigenicity. **a** GI-LM2C (*red*) and GI-LM2G (*green*) sphere cells were injected into the fourth mammary fat pad of mice with rapid tumor growth in GI-LM2G (*upper panel*) (n ≥ 3). A representative picture of mice used in this study is shown (*red arrow* = injection site for GI-LM2C; green arrow = injection site for GI-LM2G). **b** In vivo tumorigenicity assay with limited dilution; shown are the tumor-free survival curves for 10,000 (*left*), 1000 (*middle*), and 100 (*right*) injected cells (GI-LM2C (*red*) and GI-LM2G (*green*)). **c** Sections of the tumor specimens from experiments in (**a**) and (**b**) were stained for hematoxylin and eosin (H&E), Gal3 (IHC), and Gal3 IF (*to the left*, GI-LM2C, *to the right* GI-LM2G)

The consensus gold standard for cancer stemness is tumor growth in vivo following injection of serially diluted cells. Thus, we injected 2.5 × 10^5^, 1 × 10^5^, 1 × 10^4^, 1 × 10^3^, and 1 × 10^2^ GI-LM2C (red) into the right and GI-LM2G sphere-derived single cells (green) orthotopically into the left fourth mammary gland/fat pad of female, athymic nu/nu mice (Fig. 5a, right panel). Tumors of GI-LM2G cells grew significantly faster (Fig. 5a, left panel) and also at all dilutions (Fig. 5b), whereas GI-LM2C tumors grew slower, formed smaller tumors, and did not grow at the lowest dilution (Fig. 5b, right panel). Hematoxylin and eosin stain of the tumors revealed typical examples of invasive ductal carcinoma with architectural patterns, such as large sheets of tumor cells as well as cords or nests of varying size (Fig. 5c, left pictures). Immunohistochemistry (IHC, middle pictures) and immunofluorescence staining for Gal3 (right pictures) in sections of the tumor xenografts confirmed Gal3 expression in GI-LM2C-derived tumors, whereas GI-LM2G-derived tumors remained negative for Gal3 (Fig. 5c).

## Discussion

**Fig. 6:**
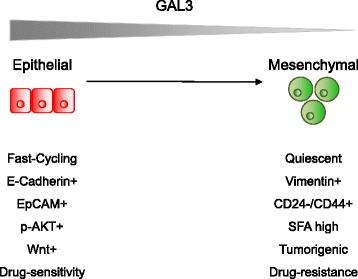
Graphical illustration of the proposed model. Model that shows the characteristics of galectin-3 (Gal3)^positive^ epithelial BCSCs (*green*, *left*) and Gal3^negative^ mesenchymal BCSCs (*red*, *right*)

Here, we present evidence that Gal3 expression is linked to an epithelial phenotype (EpCAM^+^ and E-cadherin^+^), lower drug resistance, and decreased tumorigenicity in human breast cancer cells. In contrast, Gal3^negative^ BCSCs are highly tumorigenic, phenotypically mesenchymal, drug-resistant, and enriched in BCSC marker (CD24^-^/CD44^+^)-expressing cells compared to Gal3^positive^ cells as summarized in Fig. 6. These results are surprising, because Gal3 expression has been previously associated with characteristics associated with CSCs, including tumor progression, in other cancer types [15, 18, 28–31].

The in vitro and in vivo data presented in this study are in line with TMA analysis of node-positive breast cancer patients, which revealed that patients with Gal3^negative^ tumors had significantly worse outcomes with respect to locoregional relapse-free (LRRF) survival, disease-specific survival (DSS) and overall survival (OS). Moreover, we were able to detect trends that tumors with advanced T and N stages or higher tumor grading seemed to express less Gal3. Lymphovascular invasion was significantly associated with lower Gal3 expression. These results are strengthened by analysis of data from The Cancer Genome Atlas, which also revealed lower Gal3 expression in advanced stage breast cancers.

All of these hallmarks of tumor progression have been reported to be associated with a higher BCSC pool. The BCSC pool can be expanded by epithelial–mesenchymal transition [32, 33]. Here, we were able to demonstrate in our in vitro model system that depletion of Gal3 leads to EMT with subsequent reinforcement of CSC-like features.

Despite the association of Gal3 expression in BCSCs with reduced drug resistance and low tumorigenicity in our model system, canonical Wnt signaling and AKT activity were found to be higher in Gal3^positive^ BCSCs. This is in line with previously published findings [25] and suggests a role for Gal3 as regulator of essential cancer pathways. Activity of both pathways has been linked to increased proliferation, which was evident in the epithelial Wnt- and AKT-positive BCSCs (Fig. 6). The role of Gal3 in individual processes which affect progression in breast cancer and their dynamic interactions therefore need to be further determined.

Breast cancer is not a singular, homogeneous disease, but rather a very heterogeneous malady with a variety of subtypes (basal, claudin-low, HER2+, luminal A/B, triple-negative) [34]. The patient cohort we examined represents a selected and small subset of estrogen receptor-positive and node-positive breast cancer patients who received doxorubicin-based chemotherapy, but never tamoxifen, during the course of treatment. Therefore, we cannot draw general conclusions about the role of Gal3 in all breast cancer subtypes or in node-negative, untreated patients.

It remains to be determined whether the biological and clinical behavior of other breast cancer subtypes can be characterized by Gal3 expression, and whether Gal3 might be an inducer of a subtype switch [35]. Gal3 might also be differentially expressed during the various processes involved in tumorigenesis and metastasis. It is conceivable, for instance, that Gal3 does not play a significant role in primary tumor growth, but becomes important in progression of sessile epithelial to circulating and mesenchymal tumor cells [36], protective against anoikis-induced cell death [26], and the process of endothelial attachment and subsequent extravasation.

It will be important to evaluate the expression of Gal3 in circulating tumor cells, and its role as a predictive biomarker in determining response to therapy and DSS and OS in prospective clinical trials in a variety of breast cancer patients.

## Conclusions

In summary, we present compelling evidence that in a select subgroup of breast cancers, loss of Gal3 is associated with a mesenchymal BCSC subtype and enhanced tumorigenicity, predicts poor response to chemotherapy, and therefore correlates with poor prognosis.

## Additional files

Additional file 1: Supplementary experimental procedures. (DOCX 16 kb) Additional file 2: Figure S1. The Cancer Genome Atlas (TCGA) data show the gene expression of Gal3 (*LGALS3*) in normal (no value), ductal breast carcinoma in situ and invasive ductal breast carcinoma (A) or normal (no value), primary site and metastatic site of human breast cancer samples (B). (C) Western blot analysis of whole cell lysates of GI-101A and its derivatives (GI-LM2, GI-LM2C, GI-LM2G) on estrogen receptor (ER) expression. **Figure S2** (A) Immunofluorescence staining of GI-LM2C (*upper row*) and GI-LM2G spheres (*lower row*) for Gal3 (*red*), E-cadherin (CDH1, *green*), and vimentin (*gray*). (B) Immunofluorescence staining of the same cell lines for cytokeratin 18 (*red*) and vimentin (*green*). Counterstaining with DAPI (*blue*) was used to visualize cell nuclei. **Figure S3** (A) Flow cytometric analysis shows that Gal3-positive populations (*in red*) of the same cell line consistently contain a lower BCSC pool than Gal3-negative populations (*in green*). (B) Correlation of Gal3 with CD24 and EpCAM expression is listed in a table. **Figure S4** (A) Brightfield pictures of spheres in low magnification. Figure is related with Fig. 3a. (B) Sphere-formation assay and its quantification of GI-101A, GI-101A after knockout of Gal3 (GI-101A-G) as well as derivatives GI-LM2C and GI-LM2G. (C) Western blot of whole cell lysates of GI-LM2C and GI-LM2G for Wnt targets Axin2 and Tcf4. Loading control β-actin was used. The same membrane is used in Fig. S1C. (PPTX 2636 kb)
