# Supplementary material for: Low expression of galectin-3 is associated with poor survival in node-positive breast cancers and mesenchymal phenotype in breast cancer stem cells
Source: Breast Cancer Res. 2016 Sep 29;18:97. doi: 10.1186/s13058-016-0757-6 (PMC5043623; doi:10.1186/s13058-016-0757-6)
Supplement: Additional file 1: — Supplementary experimental procedures. (DOCX 16 kb) [file 13058_2016_757_MOESM1_ESM.docx]

**Supplementary Information**

**Supplementary Experimental Procedures**

**Western blot**

Protein sample preparation and western blot analysis were carried out as described previously (29). In short, whole cell extracts were lysed in SDS lysis buffer, equal amounts of protein loaded onto 12% SDS-PAGE gels, and then transferred electrophoretically to a nitrocellulose membrane. Membranes were incubated over night with primary antibodies to Galectin-3 and after three washing steps subsequently followed by incubation with a secondary horseradish peroxidase-conjugated antibody. Enhanced chemiluminescence (ECL) reagents (Amersham Biosciences) were used for detection.

**Cell cycle proliferation and cytotoxicity assays**

Spheres were dissociated into single-cell suspension and 1X106 cells were seeded in 2ml of CSC media in 6-well low-attachment plates and allowed to recover for 24 hrs. Breast cancer cells were exposed to the FAC regimen (final concentrations of 5-FU (F) 2.5 uM, doxorubicin (A) 1ug/ml, and cyclophosphamid 0.4 ug/ml) for 24 hrs (all components were purchased from Sigma®). All cells were combined and tested for apoptosis using the Apo Alert BrdU assay (BD Bioscience, #556405) according to the manufacturer's instruction, quantified by using flow cytometry (BCI Gallios analyzer from Coulter Counter) and analyzed with the FlowJo software.

**MTT Proliferation assay**

Spheres were dissociated to single-cell suspension and 1000 cells were seeded in CSC medium in low-attachment 96 well plates. Cell growth was determined every 24 hours for 7 days by MTT [3-(4, 5-Dimethyl-2-thiazolyl-2, 5-diphenyl-2H-tetrazolium bromide)], (Promega,#G5430, CellTiter 96 Aqueous Non-Radioactive Cell Proliferation Assay kit) according to manufacturer’s instructions. Colormetric reading at 570 nm was performed using an MRX Revelation micro plate reader (Dynex). The results are expressed as the mean OD from triplicate determination.

**Flow cytometry assays**

Assays of surface expression of galectin-3 and CSC markers (CD24, CD44, CD166, EpCAM) were performed as follows: Cells were collected in Versene solutions (Invitrogen, Carlsbad, CA, USA, #15040-066), washed, and blocked with flow cytometry buffers (Santa Cruz Biotechnology) followed by labeling with 5 mg of either rat anti-galectin-3 (TIB-166 ATCC, Manassas, VA, USA) or goat anti-DR4 or mouse anti-DR5 (R&D Systems) or with the corresponding control IgG antibodies (Abcam, Cambridge, MA, USA) for 1 h on ice. Secondary antibodies used were Alexa Fluor 594-labeled donkey anti-rat, Alexa Fluor 647-labeled donkey anti-goat, or Alexa Fluor 488-labeled donkey anti-mouse, respectively (Invitrogen). For the assessment of loss of cell surface death receptors, cells were pretreated with 100 ng/ml TRAIL for 30 min at 371C followed by labeling with anti-DR4 or anti- DR5 as above. Cells were then fixed with FCM fixative buffer (Santa Cruz Biotechnology, #sc-3622) and analyzed with a XL-MCL FACS Cytometer (BD Biosciences) for samples labeled with Alexa Fluor 488, or Fortessa FACS cytometer (BD Biosciences) for samples stained with Alexa Fluor 647, and FlowJo version 8.8.6 acquisition/analysis software (BD Biosciences).

**Supplementary Figure Legends**

**Figure S1:**

The Cancer Genome Atlas (TCGA) data show the gene expression of Gal3 (*LGALS3*) in normal (no value), ductal breast carcinoma in situ and invasive ductal breast carcinoma (A) or normal (no value), primary site and metastatic site of human breast cancer samples (B). (C) Western blot analysis of whole cell lysates of GI-101A and its derivatives (GI-LM2, GI-LM2C, GI-LM2G) on estrogen receptor (ER) expression.

**Figure S2:**

(A) Immunofluorescence staining of GI-LM2C (upper row) and GI-LM2G spheres (lower row) for Gal3 (red), E-Cadherin (CDH1, green), and Vimentin (grey). (B) Immunofluorescence staining of the same cell lines for cytokeratin 18 (red) and Vimentin (green). Counterstaining with DAPI (blue) was used to visualize cell nuclei.

**Figure S3:**

(A) Flow cytometric analysis shows that Gal3 positive populations (in red) of the same cell line consistently contain a lower BCSC pool than Gal3 negative populations (in green). (B) Correlation of Gal3 with CD24 and EpCAM expression is listed in a table.

**Figure S4:**

(A) Brightfield pictures of spheres in low magnification. Figure is related with Fig. 3A. (B) Sphere-formation assay and its quantification of GI-101A, GI-101A after knock-out of Gal3 (GI-101A-G) as well as derivatives GI-LM2C and GI-LM2G. (C) Western blot of whole cell lysates of GI-LM2C and GI-LM2G for Wnt targets Axin2 and Tcf4. Loading control β-Actin was used. The same membrane is used in Fig. S1C.
