# Supplementary material for: Low expression of galectin-3 is associated with poor survival in node-positive breast cancers and mesenchymal phenotype in breast cancer stem cells
Source: Breast Cancer Res. 2016 Sep 29;18:97. doi: 10.1186/s13058-016-0757-6 (PMC5043623; doi:10.1186/s13058-016-0757-6)
Supplement: Additional file 2: Figure S1. — The Cancer Genome Atlas (TCGA) data show the gene expression of Gal3 (LGALS3) in normal (no value), ductal breast carcinoma in situ and invasive ductal breast carcinoma (A) or normal (no value), primary site and metastatic site of human breast cancer samples (B). (C) Western blot analysis of whole cell lysates of GI-101A and its derivatives (GI-LM2, GI-LM2C, GI-LM2G) on estrogen receptor (ER) expression. Figure S2 (A) Immunofluorescence staining of GI-LM2C (upper row) and GI-LM2G spheres (lower row) for Gal3 (red), E-cadherin (CDH1, green), and vimentin (gray). (B) Immunofluorescence staining of the same cell lines for cytokeratin 18 (red) and vimentin (green). Counterstaining with DAPI (blue) was used to visualize cell nuclei. Figure S3 (A) Flow cytometric analysis shows that Gal3-positive populations (in red) of the same cell line consistently contain a lower BCSC pool than Gal3-negative populations (in green). (B) Correlation of Gal3 with CD24 and EpCAM expression is listed in a table. Figure S4 (A) Brightfield pictures of spheres in low magnification. Figure is related with Fig. 3a. (B) Sphere-formation assay and its quantification of GI-101A, GI-101A after knockout of Gal3 (GI-101A-G) as well as derivatives GI-LM2C and GI-LM2G. (C) Western blot of whole cell lysates of GI-LM2C and GI-LM2G for Wnt targets Axin2 and Tcf4. Loading control β-actin was used. The same membrane is used in Fig. S1C. (PPTX 2636 kb) [file 13058_2016_757_MOESM2_ESM.pptx]

## Slide 1
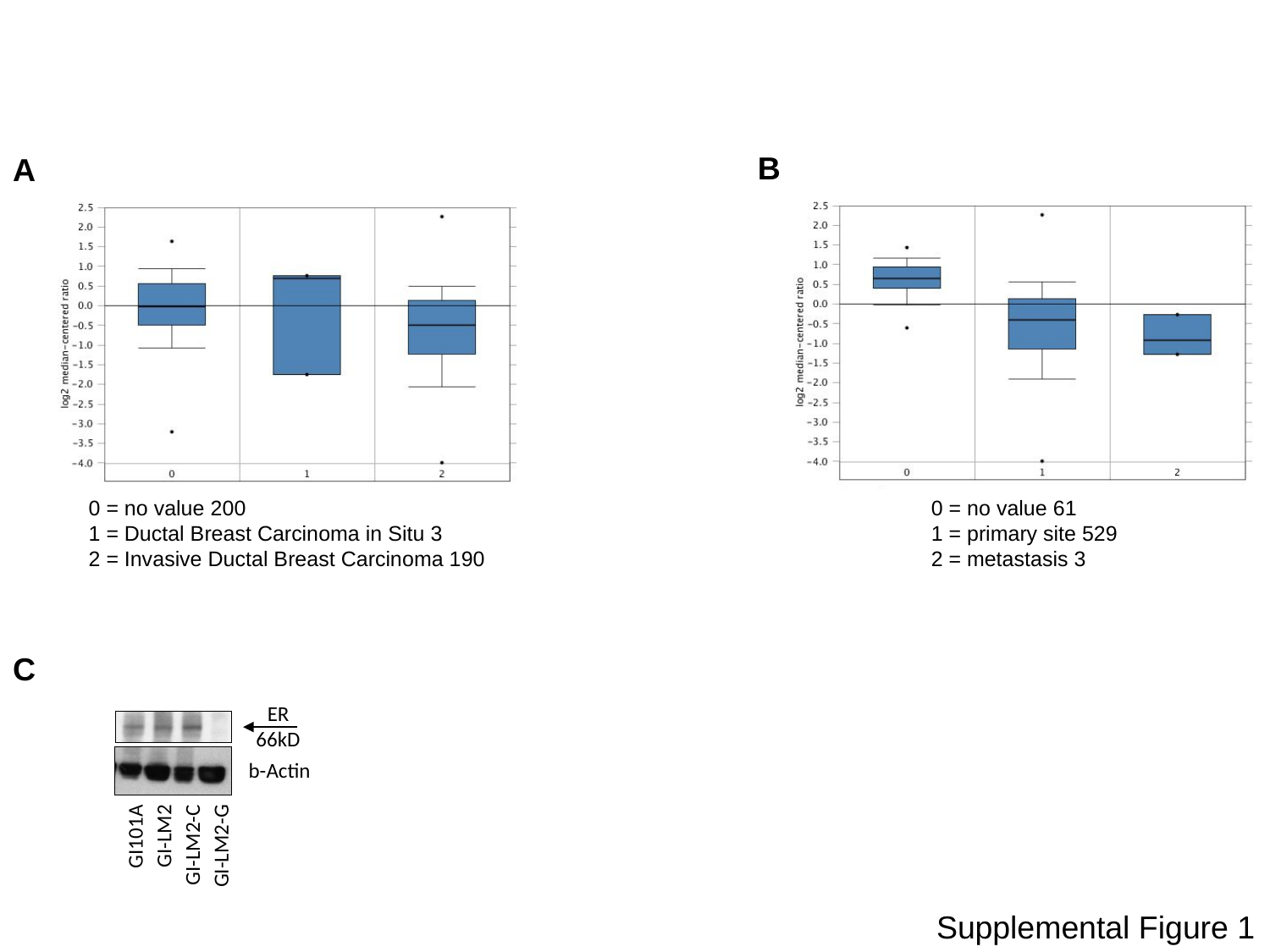

B
A
0 = no value 200
1 = Ductal Breast Carcinoma in Situ 3
2 = Invasive Ductal Breast Carcinoma 190
0 = no value 61
1 = primary site 529
2 = metastasis 3
C
ER
66kD
b-Actin
GI-LM2
GI101A
GI-LM2-C
GI-LM2-G
Supplemental Figure 1

## Slide 2
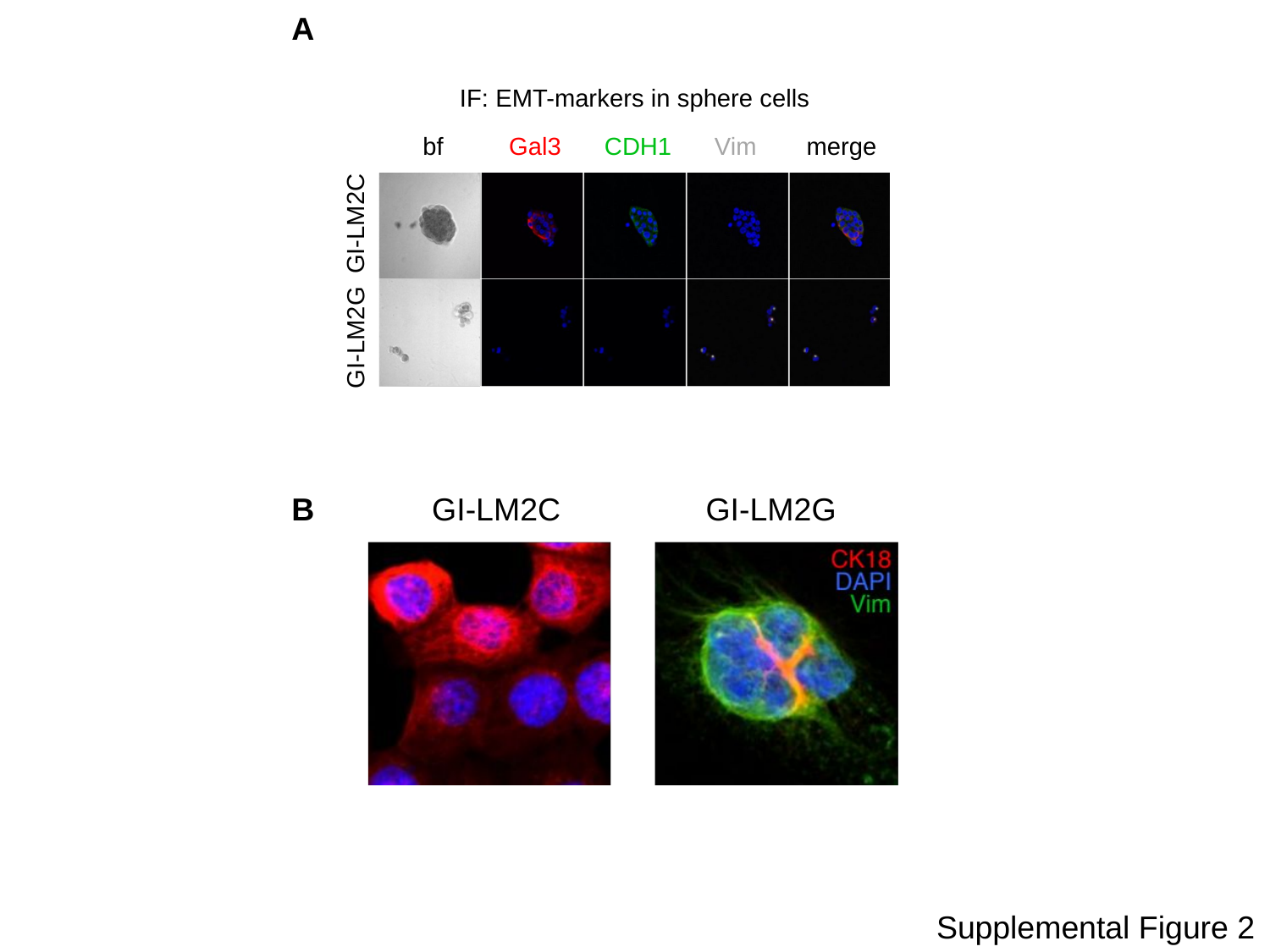

A
IF: EMT-markers in sphere cells
bf
Gal3
CDH1
Vim
merge
GI-LM2C
GI-LM2G
GI-LM2C
GI-LM2G
B
Supplemental Figure 2

## Slide 3
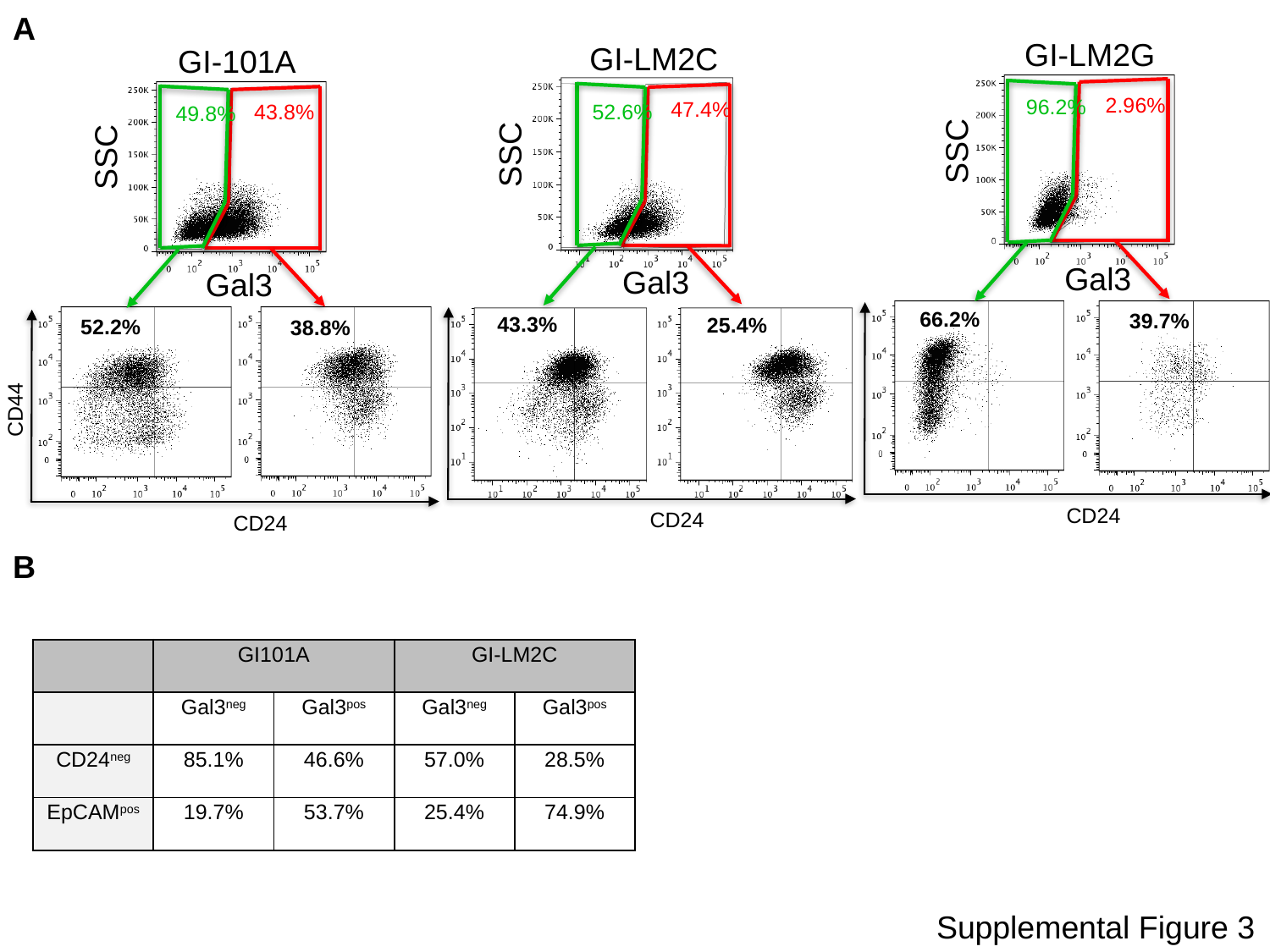

A
GI-LM2G
2.96%
96.2%
SSC
Gal3
66.2%
39.7%
CD44
CD24
GI-LM2C
47.4%
52.6%
SSC
Gal3
43.3%
25.4%
CD24
GI-101A
43.8%
49.8%
SSC
Gal3
52.2%
38.8%
CD44
CD24
B
| | GI101A | | GI-LM2C | |
| --- | --- | --- | --- | --- |
| | Gal3neg | Gal3pos | Gal3neg | Gal3pos |
| CD24neg | 85.1% | 46.6% | 57.0% | 28.5% |
| EpCAMpos | 19.7% | 53.7% | 25.4% | 74.9% |
Supplemental Figure 3

## Slide 4
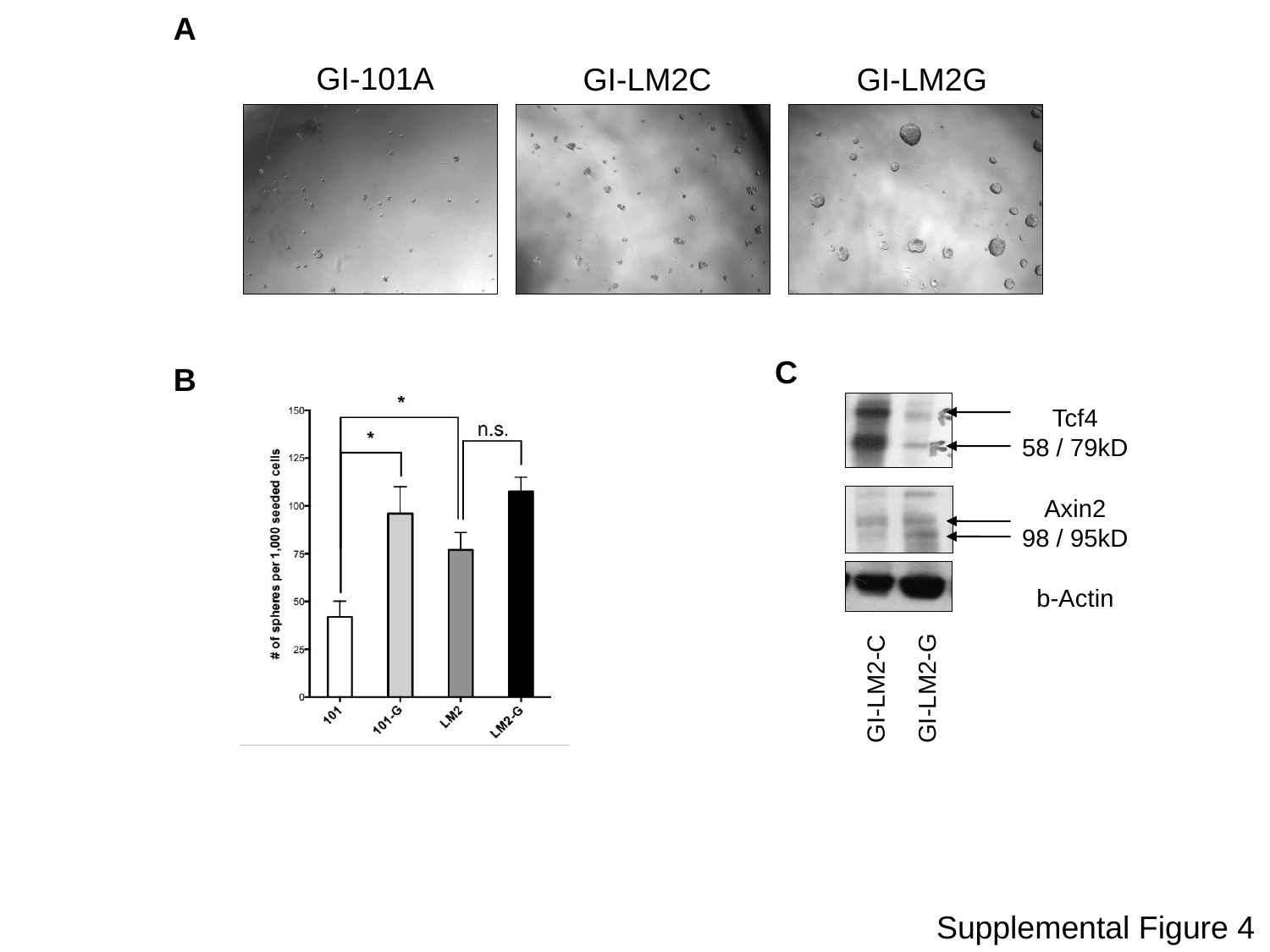

A
GI-101A
GI-LM2C
GI-LM2G
C
B
Tcf4
58 / 79kD
Axin2
98 / 95kD
b-Actin
GI-LM2-G
GI-LM2-C
Supplemental Figure 4
